# Evidence for Shared Cognitive Processing of Pitch in Music and Language

**DOI:** 10.1371/journal.pone.0073372

**Published:** 2013-08-15

**Authors:** Tyler K. Perrachione, Evelina G. Fedorenko, Louis Vinke, Edward Gibson, Laura C. Dilley

**Affiliations:** 1 Department of Brain and Cognitive Sciences, Massachusetts Institute of Technology, Cambridge, Massachusetts, United States of America; 2 Department of Psychology, Bowling Green State University, Bowling Green, Ohio, United States of America; 3 Department of Communicative Sciences and Disorders, Michigan State University, East Lansing, Michigan, United States of America; CSIC-Univ Miguel Hernandez, Spain

## Abstract

Language and music epitomize the complex representational and computational capacities of the human mind. Strikingly similar in their structural and expressive features, a longstanding question is whether the perceptual and cognitive mechanisms underlying these abilities are shared or distinct – either from each other or from other mental processes. One prominent feature shared between language and music is signal encoding using pitch, conveying pragmatics and semantics in language and melody in music. We investigated how pitch processing is shared between language and music by measuring consistency in individual differences in pitch perception across language, music, and three control conditions intended to assess basic sensory and domain-general cognitive processes. Individuals’ pitch perception abilities in language and music were most strongly related, even after accounting for performance in all control conditions. These results provide behavioral evidence, based on patterns of individual differences, that is consistent with the hypothesis that cognitive mechanisms for pitch processing may be shared between language and music.

## Introduction

The production and perception of spoken language and music are two distinctly human abilities exemplifying the computational and representational complexities of the human mind. These abilities appear to be both unique to our species and universal across human cultures, and scholars have speculated at length about the extent to which these abilities are related [1,2]. Language and music superficially appear to share many features, including most prominently hierarchical structural organization [3-6], the ability to convey meaningful content and reference [7,8], and the encoding of learned categories via shared perceptual/motor systems [9]. The prevalence of such high-level, abstract similarities has led some to suggest that music is parasitic on language [10,11] or *vice versa* [12], although evidence from brain-damaged individuals [e.g., 13], as well as recent neuroimaging studies [e.g., 14-16], challenge the link between language and music at the level of structural processing.

One domain in which the similarities between language and music have led to specific proposals of shared mechanisms is that of pitch perception. Pitch is a core component of spoken language, helping to disambiguate syntactic structures [17-19] and to convey both pragmatic and semantic meaning [20,21]. In music, relative pitch changes convey melodic structure, whether played on instruments or sung by voice. Research in cognitive psychology and neuroscience has suggested that pitch processing in language and music may rely on shared mechanisms. In the auditory brainstem, linguistic pitch patterns are encoded with higher fidelity in musicians than non-musicians [22]. Expert musicians are better able to perceive spoken language in the presence of background noise [23], a process that is thought to depend in part on following the pitch pattern of an attended voice [24]. Individuals with more extensive musical training are better able to learn a foreign language that uses pitch specifically as a phonological contrast [25], and individuals with greater musical aptitude demonstrate greater proficiency with second-language phonological processing generally [26]. Listeners exhibiting musical tone-deafness (amusia) are also likely to be impaired in their ability to make linguistic distinctions on basis of pitch [27-30].

However, the existing evidence for shared pitch processing mechanisms in language and music is not without caveats. Many studies focus on expert musicians, who may represent an exceptional case not generalizable to the population at large [31-33]. Studies that relate pitch processing in language and music on the basis of the frequency-following response in brainstem electrophysiology are measuring a preattentive sensory response to the fundamental frequency of sounds, prior to any conscious pitch percept or distinction between language and music in the cortex. Behaviorally, the categorical use of pitch differs between language, where pitch varies continuously and is normalized with respect to the range of an individual speaker [34,35], and music, where pitch is encoded as musical notes, often with discrete frequencies, which are represented in both relative (i.e., “key”) as well as absolute terms [36]. Some of the evidence for shared pitch processing mechanisms between language and music can be explained without postulating that any shared cognitive/neural machinery be specialized for these abilities. For example, these abilities may co-vary due to their mutual reliance on the same low-level sensory pathways encoding auditory information or the same domain-general processes of attention, working memory, or motivation. Finally, some evidence even suggests that pitch processing in language and music may be supported by distinct mechanisms. Brain imaging studies of pitch perception distinguish left-lateralized linguistic pitch processing for semantic content versus right-lateralized processing of musical melody or sentence prosody [37,38, cf. 39], suggesting that transfer between musical ability and language phonology may rely on the enhancement of sensory pathways for pitch, rather than shared cognitive mechanisms *per se*. Brain injuries may impair language but leave music intact, and *vice versa* [40].

We evaluate the hypothesis that pitch processing in language and music is shared above and beyond these abilities’ mutual reliance on low-level sensory-perceptual pathways or domain-general processes like attention, working memory, and motivation. To address this question, we investigate whether pitch processing abilities in a language task are more closely related to pitch processing abilities in a music task, compared to several control tasks. In two experimental conditions, we assessed individual differences in listeners’ ability to detect subtle changes in pitch in both musical (short melodies) and linguistic (sentential prosody) contexts using designs adapted from perceptual psychophysics. We also tested individuals’ perceptual abilities in three control conditions: (1) a non-linguistic, non-musical test of psychophysical pitch discrimination threshold, designed to control for basic sensory acuity in pitch discrimination; (2) a test of temporal frequency discrimination, designed to control for basic (non-pitch) auditory perceptual acuity; and (3) a test of visual spatial frequency discrimination, designed to control for individual differences in attention and motivation. Previous work has demonstrated a variety of relationships among individual differences in both low-level auditory abilities and domain-general cognitive factors [e.g., 23,41-46]. As such, positive correlations can reasonably be expected among all five conditions, both experimental and control [47,48]; however, it is the pattern of the relative strengths of these correlations that will be most informative about the relationship between pitch perception in music and language. We hypothesized that a significant and strong relationship between these two tasks would remain after controlling for these sensory and domain-general factors. That is, we expect that the relationship between pitch perception in language and music are similar in ways that cannot be accounted for only by shared sensory acuity or domain-general resources like attention and working memory.

## Method

We measured discrimination accuracy, perceptual sensitivity, and discrimination thresholds in linguistic and musical contexts, and in three control conditions (auditory spectral frequency, auditory temporal frequency, and visual spatial frequency) designed to account for general auditory acuity and domain-general cognitive factors.

### Participants

Native English-speaking young-adult participants (*N* = 18) participated in this study. All individuals were recruited from the local university community and provided informed, written consent to participate. This study was approved by the Bowling Green State University Institutional Review Board (PI: L.D.). Participants reported no speech, hearing, language, psychological or neurological disorders, and demonstrated normal hearing by passing pure-tone audiometric screening in each ear at octave frequencies from 0.5–4.0 kHz. Participants provided information about their musical and foreign language experience via self-report (Table 1). The self-report instrument and participants’ summarized responses are available online (Archive S1).

**Table 1:** Musical and linguistic background of participants (by self-report).

**Factor**	**Count**	**Min-Max**	**Median**	**Mean**	**Std. Dev.**	**Responding N =**
Ever played an instrument	15					18
-- Number of instruments played		0 - 4	2	1.56	1.15	18
-- Years played*		0 - 17	5	5.50	5.39	18
-- Proficiency* †		0 - 10	6	5.00	3.57	18
Ever sung in a choir	12					18
-- Years in choir		0 - 14	2	3.83	4.60	18
Ever had formal music lessons	14					18
-- Years of lessons*		1-10	5	4.79	2.97	14
-- Years since last lesson*		0 - 8	3	4.36	2.98	14
-- Years since last practice*		0 - 8	1	2.39	2.97	14
Ever had formal training in music theory	6					18
-- Years of music theory training		1-11	4.5	5.33	3.39	6
Formal degree in music	1					18
Hours of music listening daily		0.75-18	3	4.43	4.43	18
Ever studied a foreign language	15					18
-- Number of foreign languages studied		1-2	1	1.33	0.49	15
-- Age foreign language study began		6-16	14	13.07	2.52	15
-- Speaking proficiency* †		1-8	3.5	4.00	2.25	15
-- Understanding proficiency* †		1-9	5	4.79	2.55	15
-- Reading proficiency* †		1-10	5	4.71	2.61	15
-- Writing proficiency* †		0 - 10	5	4.20	3.00	15

* For most proficient musical instrument or foreign language

† Scale 0 (least proficient) to 10 (most proficient)

### Stimuli

#### Language

**Figure 1:**
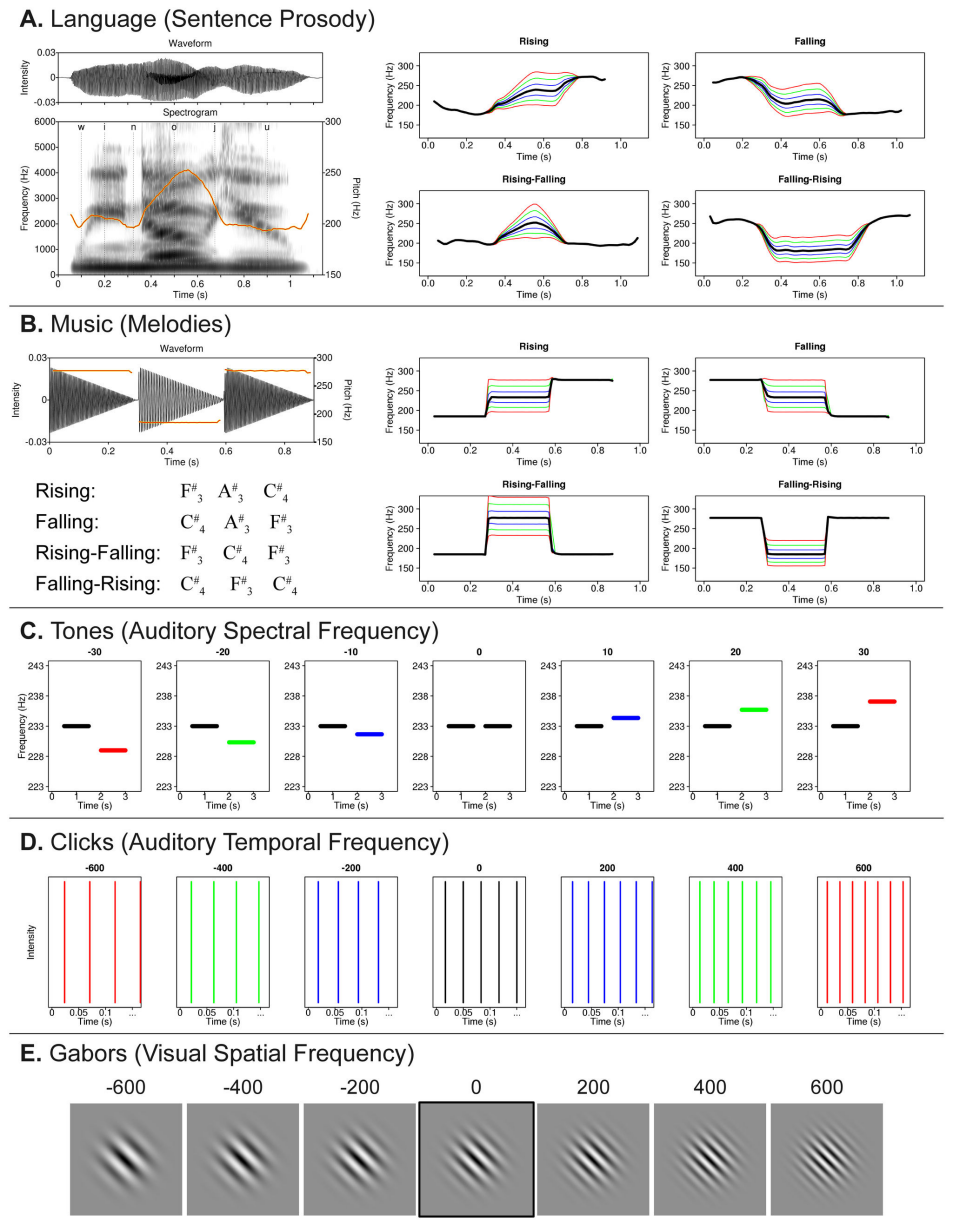
Example psychophysical stimuli. (**A**) At left, a waveform and spectrogram illustrate an example template linguistic stimulus with overlaid pitch contour (orange) and phonemic alignment. Plots at right illustrate the four different types of linguistic pitch contours (black traces) showing ±100, 200, and three hundred cents deviants (blue, green, and red traces, respectively). (**B**) At left, a waveform illustrates an example template musical stimulus with overlaid pitch contour (orange), as well as the notation of musical stimuli. Plots at right illustrate the four different types of musical pitch contours (black traces), analogous to those from the Language condition, as well as traces of deviants of ±100 (blue), 200 (green), and 300 (red) cents. (**C**) These plots show the relative frequencies of the template (black traces) and deviants of ±10 (blue), 20 (green), and 30 (red) cents, each shown within the the temporal configuration of a single trial. (**D**) These plots show the relative rates of the template click train (black lines) and rate deviants of ±200 (blue), 400 (green), and 600 (red) cents. Note that only the first 150ms of the full 1s stimuli are shown. (**E**) Visual spatial frequency stimuli (“Gabor patches”), with the template (outlined) and example deviants of ±200, 400, and six hundred cents.

An adult native English-speaking female was recorded saying the sentence “We know you,” which consists of only sonorous segments and has continuous pitch. Four natural intonation contours (1.1s in duration) were elicited for recording: rising, falling, rising-falling, and falling-rising, with approximately level pitch on each syllable (Figure 1A). These “template” stimuli were resynthesized in Praat (http://www.fon.hum.uva.nl/praat/) [49] using the pitch synchronous overlap-and-add algorithm [50] to produce “deviants”, in which the pitch of the middle syllable varied from the template by ±20-300 cents in steps of twenty cents, where one cent = one hundredth of a semitone, a ratio of 2^1/1200^ (The values for deviant stimuli for each of the five conditions were determined based on pilot experiments conducted to ensure participants’ discrimination thresholds would fall in approximately the middle of the stimulus range). These and all other auditory stimuli were normalized for RMS amplitude to 54dB SPL.

#### Music

The same four pitch contours were synthesized as three-note musical melodies (0.9s in duration) using Praat. Each 300ms note had an instantaneous rise and linear fall time (Figure 1B). The template contours consisted of the following notes: F^#^_3_, A^#^_3_, C^#^_4_ (rising); C^#^_4_, A^#^_3_, F^#^_3_ (falling); F^#^_3_, C^#^_4_, F^#^_3_ (rising-falling); C^#^_4_, F^#^_3_, C^#^_4_ (falling-rising); paralleling the pitch contours of the linguistic stimuli. These template contours were resynthesized in Praat to produce deviants, in which the pitch of the middle note varied by ±20-300 cents (in steps of twenty cents).

#### Auditory spectral frequency (Tones)

A sinusoidal pure-tone 233Hz template stimulus (1.0s in duration), as well as 30 deviant stimuli of ±2-30 cents (in steps of two cents), were synthesized using Praat. The frequency of the template stimulus was the same as the long-term average frequency of the linguistic and musical stimuli (A^#^_3_).

*Auditory temporal frequency (Clicks). S*eries of broadband clicks were synthesized using Praat. Impulses in the template click train occurred at a rate of 30Hz and totaled 1.0s in duration. Click trains with rates varying by ±40-600 cents (in steps of forty cents) were synthesized as deviants. These stimuli were band-pass filtered from 2–4kHz, with 1kHz smoothing. The design of these stimuli followed those that elicit a percept of “acoustic flutter” and are used to assess temporal processing in the auditory system distinctly from pitch [51-53].

#### Visual spatial frequency (Gabors)

The template stimulus consisted of a 360×360 pixel sinusoidal luminance grating over the full contrast range with a period of 40 pixels, rotated 45^°^ from vertical, and multiplied by a two-dimensional Gaussian envelope centered on the midpoint of the image with a standard deviation of 0.375 (135 pixels) and a baseline luminance of 50%. Luminance grating deviants, in which spatial frequencies varied from the template by ±40-600 cents (in steps of forty cents), were similarly generated using custom MATLAB code (Mathworks, Natick, MA).

For each condition, the Praat and MATLAB scripts used to generate the stimuli (Archive S2) and the stimuli themselves (Archive S3) are available online.

### Procedure

Participants completed seven self-paced experimental sessions counterbalanced using a Latin-square design (the Music and Language conditions were each divided into two sessions to reduce their length, one consisting of the rising and falling-rising contours, the other consisting of the falling and rising-falling contours). All stimuli were delivered using E-Prime 1.1 (Psychology Software Tools, Sharpsburg, PA) via a PC-compatible computer with a Dell 19″ UltraSharp 1907FP Flat Panel VGA/DVI monitor at a resolution of 1024×768 pixels and 16-bit color depth and a Creative Sound Blaster Audigy SE soundcard in a quiet room over Sennheiser HD-280 Pro headphones. Participants’ task in all five conditions was to indicate whether two stimuli in a pair were the same or different.

In all conditions, each trial consisted of the template stimulus followed by a brief inter-stimulus interval (ISI) and then either a deviant stimulus (75% of the trials) or the repeated template (25% of the trials). Each magnitude of deviant stimuli (e.g., ±20-300 cents for the Language condition) occurred equally frequently, and the presentation order was randomized. Participants indicated their response by button press. A brief inter-trial interval (ITI) preceded the presentation of the next template stimulus. Prior to each condition, participants were familiarized with the task through 14 practice trials (6 “same” trials) with corrective feedback.

#### Language and Music

These conditions were assessed in two sessions each, consisting of 240 trials blocked by contour. In these conditions, ISI was 750ms and the ITI was 1.0s. Each of the four language and music sessions lasted approximately 20 minutes, and participants were offered a short break after every 40 trials. Deviant stimuli in the practice trials were ±140 or three hundred cents.

#### Auditory spectral frequency (Tones)

This session consisted of 240 trials lasting all together approximately 14 minutes. The ISI and ITI were both 500ms. Participants were offered a short break after 120 trials. Deviant stimuli in the practice trials were ±14 or thirty cents.

#### Auditory temporal frequency (Clicks)

This session consisted of 240 trials lasting all together approximately 14 minutes. The ISI and ITI were both 500ms. Participants were offered a short break after 120 trials. Deviant stimuli in the practice trials were ±280 or six hundred cents.

#### Visual spatial frequency (Gabors)

This session consisted of 240 trials lasting all together approximately 14 minutes. In this condition, each stimulus was presented for 1s, ISI was 500ms, and the ITI was 750ms. During the ISI and ITI, the screen was blank (50% luminance). Participants were offered a short break after 120 trials. Deviant stimuli in the practice trials differed from the standard by ±280 or six hundred cents in spatial frequency. During this condition, participants’ heads were situated securely in a chin rest, with eyes a fixed distance from the monitor to ensure stimuli occupied a consistent visual angle both across trials and across subjects.

## Results

### Accuracy, Sensitivity, and Thresholds

**Figure 2:**
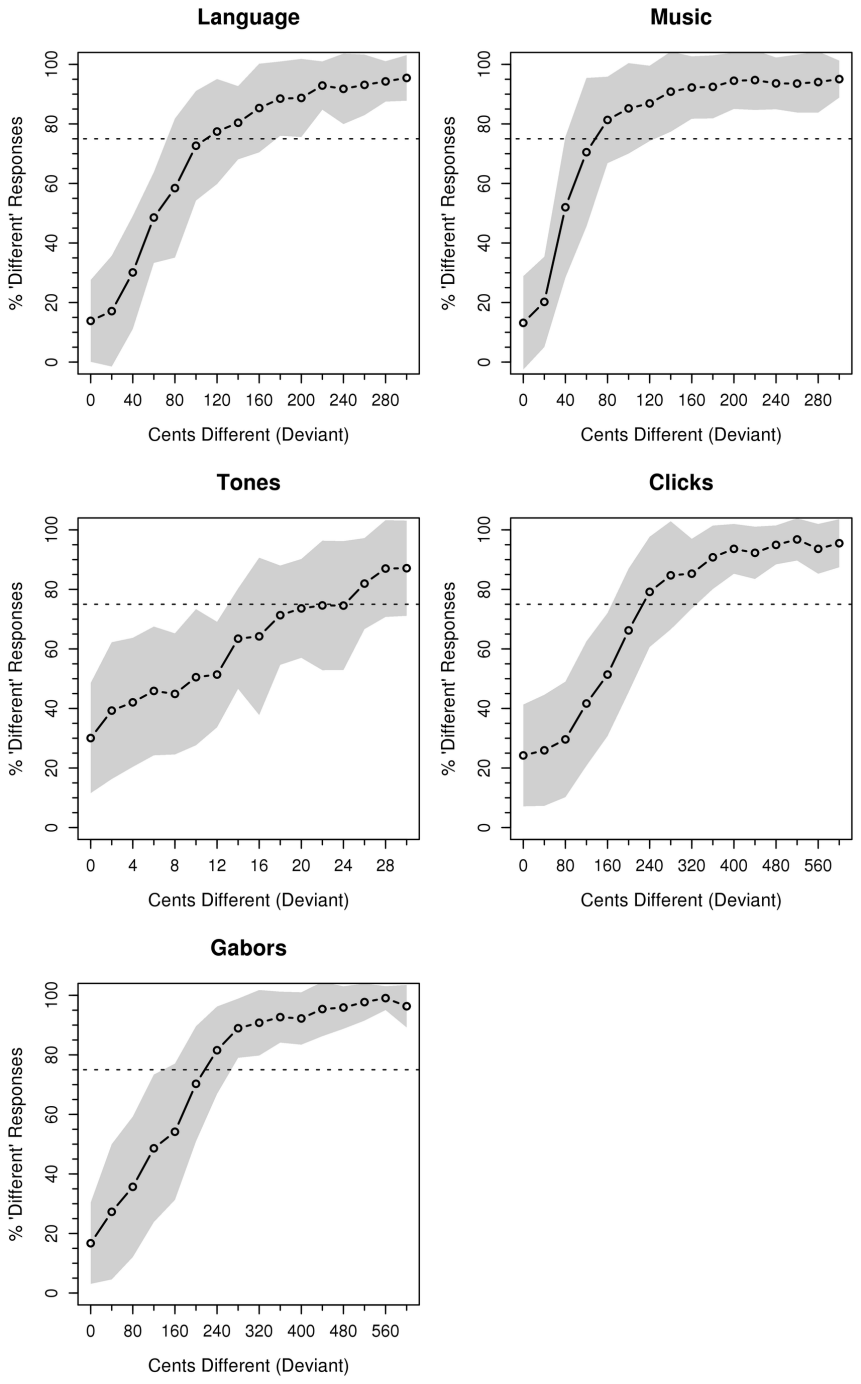
Discrimination contours across stimulus conditions. Mean percent "different" responses are shown for each condition (note differences in abscissa values). Shaded regions show the standard deviation of the sample. Dotted horizontal line: 75% discrimination threshold. Ordinate: frequency of “different” responses; Abscissa: cents different from the template.

We assessed participants’ performance on the five tasks through three dependent measures: accuracy (percent correct responses), sensitivity (*A*') [54], and threshold (physical difference in stimuli at and above which participants exceeded 75% discrimination accuracy). Table 2 delineates the overall mean and distribution of participant performance on these measures, and Figure 2 shows the discrimination contours. Measured values for pure-tone discrimination threshold (26 ± 5 cents) versus a reference tone of 233 Hz closely correspond to previously reported values in this range [33,55]. Participants’ aggregated results are available online (Archive S4).

**Table 2:** Task performance by condition.

	**OverallAccuracy**		**Sensitivity(*A*')**		**Threshold(cents)**	
**Conditions**	**Mean**	**Std. Dev.**	**Mean**	**Std. Dev.**	**Mean**	**Std. Dev.**
Language	0.77	± 0.09	0.87	± 0.08	151	± 74
Music	0.83	± 0.09	0.90	± 0.08	129	± 92
Tones	0.65	± 0.09	0.75	± 0.10	26	± 5
Clicks	0.75	± 0.06	0.84	± 0.07	313	± 137
Gabors	0.79	± 0.08	0.88	± 0.07	296	± 143

We employed a series of pairwise correlations and multiple linear regression models (using **R**, v 2.15.2, http://www.r-project.org/) to address the hypothesis that pitch processing in language and music relies on shared mechanisms. Differences in average performance between the various conditions are immaterial to this hypothesis, given that such values are partially a function of the range of physical stimulus differences we selected for each condition. The question of whether pitch processing mechanisms are shared is best addressed through modeling the shared variance among the tasks – that is, the extent to which individual differences in performance are consistent across conditions.

### Pairwise correlations

We assessed the null hypothesis that participants’ performance on each of our five stimulus categories was independent of their performance on the other conditions through a series of pairwise Pearson’s product-moment correlations (Table 3). We adopted a significance criterion of α = 0.05 and, following Bonferroni correction for 30 tests (10 condition pairs and 3 dependent measures), correlations with *p* < 0.00167 were considered statistically significant.

**Table 3:** Pairwise correlations.

		**OverallAccuracy**		**Sensitivity(*A*')**		**Threshold**	
**Conditions**		***r* =**	***p* <**	***r=***	***p<***	***r* =**	***p* <**
**Language**	Music	0.927	0.000*	0.928	0.000*	0.905	0.000*
	Tones	0.780	0.000*	0.732	0.001*	0.601	0.008
	Clicks	0.749	0.000*	0.713	0.001*	0.661	0.003
	Gabors	0.389	0.111	0.576	0.012	0.562	0.015
**Music**	Tones	0.671	0.002	0.642	0.004	0.522	0.026
	Clicks	0.626	0.005	0.558	0.016	0.684	0.002
	Gabors	0.374	0.126	0.506	0.032	0.550	0.018
**Tones**	Clicks	0.752	0.000*	0.687	0.002*	0.330	0.180
	Gabors	0.384	0.115	0.559	0.016	0.540	0.021
**Clicks**	Gabors	0.425	0.079	0.466	0.051	0.543	0.020

* significant at Bonferroni-corrected α = 0.00167

A number of pairwise correlations reached significance. Importantly, only the correlation between Language and Music was significant across all three dependent measures. Moreover, participants’ performance in the Music condition was not significantly correlated with any other condition besides Language.

For each dependent measure, the correlation between performance on the Language and Music conditions was compared against the next strongest correlation between either of these and a third condition [56,57]. For overall accuracy, the correlation between Language and Music was significantly stronger than the next best correlation (Language and Tones; *z* = 1.98, *p* < 0.025). For sensitivity (*A*'), the correlation between Language and Music was significantly stronger than the next best correlation (Language and Tones; *z* = 2.32, *p* < 0.011). Finally, for discrimination threshold, the correlation between Language and Music was again significantly stronger than the next best correlation (Music and Clicks, *z* = 2.23, *p* < 0.013).

### Linear models

Because pairwise correlations suggested multiple dependency relationships among the five stimulus categories, we next employed a series of multiple linear regression models to examine whether participants’ abilities in the Language and Music conditions were related above and beyond the differences in performance explained by the control conditions. For each of the three dependent measures, we constructed a pair of nested linear models: In the first of these models (the reduced model), performance in the condition of interest (Language or Music) was accounted for with respect to the three control conditions. In the second model (the full model), the other condition of interest (Music or Language, respectively) was added to the model. These linear models are summarized in Table 4 and Table 5.

**Table 4:** Comparison of linear models of language performance.

	**Model**		**Terms**				
**OverallAccuracy**	***R*^2^ =**	***p* <**		**Tones**	**Clicks**	**Gabors**	**Music**
Language ~ Tones + Clicks + Gabors	0.671	0.001	β =	0.495	0.505	0.054	−
			*p* =	0.054	0.155	0.786	−
Language ~ Tones + Clicks + Gabors + Music	0.919	6×10^-7^	β =	0.181	0.261	-0.023	0.655
			*p* =	0.191	0.165	0.822	3×10^-5^
**Sensitivity(*A*')**							
Language ~ Tones + Clicks + Gabors	0.646	0.002	β =	0.302	0.435	0.229	−
			*p* =	0.139	0.119	0.323	−
Language ~ Tones + Clicks + Gabors + Music	0.923	5×10^-7^	β =	0.065	0.273	0.071	0.696
			*p =*	0.525	0.055	0.533	2×10^-5^
**Threshold**							
Language ~ Tones + Clicks + Gabors	0.606	0.004	β =	0.636	0.520	0.090	−
			*p* =	0.068	0.030	0.706	−
Language ~ Tones + Clicks + Gabors + Music	0.846	4×10^-5^	β =	0.287	0.092	0.009	0.599
			*p* =	0.220	0.595	0.956	6×10^-4^

**Table 5:** Comparison of linear models of music performance.

	**Model**		**Terms**				
**OverallAccuracy**	***R*^2^ =**	***p* <**		**Tones**	**Clicks**	**Gabors**	**Language**
Music ~ Tones + Clicks + Gabors	0.492	0.021	β =	0.480	0.372	0.118	−
			*p* =	0.146	0.417	0.655	−
Music ~ Tones + Clicks + Gabors + Language	0.874	1×10^-5^	β =	-0.090	-0.208	0.056	1.149
			*p* =	0.635	0.415	0.682	3×10^-5^
**Sensitivity(*A*')**							
Music ~ Tones + Clicks + Gabors	0.463	0.030	β =	0.340	0.233	0.227	−
			*p* =	0.185	0.495	0.437	−
Music ~ Tones + Clicks + Gabors + Language	0.883	7×10^-6^	β =	0.001	-0.255	-0.030	1.123
			*p* =	0.992	0.172	0.837	2×10^-5^
**Threshold**							
Music ~ Tones + Clicks + Gabors	0.572	0.007	β =	0.582	0.714	0.135	−
			*p* =	0.185	0.023	0.662	−
Music ~ Tones + Clicks + Gabors + Language	0.832	6×10^-5^	β =	-0.063	0.186	0.044	1.015
			*p* =	0.841	0.406	0.826	6×10^-4^

To determine whether the full model better explained the range of performance in the condition of interest, each pair of full and reduced models were compared using an analysis of variance. On all dependent measures, the full models including both the Music and Language conditions explained significantly more variance than the reduced models consisting of only the control conditions [Overall Accuracy: *F*_1,13_ = 39.60, *p* = 3×10^-5^; Sensitivity (*A*'): *F*_1,13_ = 46.48, *p* = 2×10^-5^; Threshold: *F*_1,13_ = 20.18, *p* = 0.0006]. For all three dependent measures, there remained a significant relationship between participants’ performance in the Language and Music conditions even after controlling for the effect of the three control conditions. That is, individual differences in processing music and language rely on additional shared processes beyond the low-level sensory and domain-general cognitive abilities assessed by these three control tasks.

### Perceptual Abilities and Musical Background

Some relationships were observed between participants’ self-reported musical background and their performance on the perceptual tasks. Participants who had played an instrument for a greater amount of time tended to perform better in the Music condition (Accuracy: *r* = 0.672, *p* < 0.0023; Sensitivity (A'): *r* = 0.568, *p* < 0.014; Threshold: *r* = -0.588, *p* < 0.011), although this effect was only marginal in the Language condition (Accuracy: *r* = 0.471, *p* < 0.05; Sensitivity (A'): *r* = 0.439, *p* = 0.07; Threshold: *r* = -0.460, *p* = 0.055); however, it was not observed for any of the control conditions. The more recently that participants reported having practiced an instrument, the better they tended to perform in the Music condition (Accuracy: *r* = -0.769, *p* < 0.0014; Sensitivity (A'): *r* = -0.823, *p* < 0.0003; Threshold: *r* = 0.779, *p* < 0.0011) and in the Language condition (Accuracy: *r* = -0.687, *p* < 0.0014; Sensitivity (A'): *r* = -0.792, *p* < 0.00075; Threshold: *r* = 0.561, *p* < 0.037), but this effect was not observed in any of the control conditions. No other self-reported measure was reliably associated with performance on the psychophysical tasks.

## Conclusions

After controlling for their performance on the three control tasks, the persistent relationship between participants’ ability to discriminate differences in linguistic pitch (sentence prosody) and musical pitch (melodies) is consistent with the hypothesis that cognitive mechanisms for pitch processing in language and music are shared beyond simple reliance on overlapping auditory sensory pathways or domain-general working memory and attention. There exists a significant and strong relationship between individuals’ pitch processing abilities in music and language. Such a relationship remains even after controlling for individuals’ performance on a range of control tasks intended to account for basic non-linguistic and non-musical sensory acuity for pitch, as well as domain-general mnemonic, attentional, and motivational factors that bear on laboratory tests of perception. Importantly, this higher-order relationship between linguistic and musical pitch processing was observed in participants drawn from the general population, rather than a sample selected specifically for musical expertise or neurological deficit affecting speech or music.

The persistent relationship between pitch processing in language and music beyond what can be explained by these three control tasks does not preclude the possibility that other domain general processes, whether perceptual or cognitive, may eventually be enumerated to account for the remaining variance. Although we excluded auditory acuity for pitch (tones), non-pitch auditory acuity (clicks) and general attention and motivation for psychophysical tasks (gabors), there may exist other factors that contribute to the remaining shared variance between language and music. For example, although previous studies have not found relationships between indices of higher-level cognitive processes (such as IQ or working memory) and lower-level auditory perception [44], it may be the case that these psychometric factors bear on linguistic and musical pitch processing after sensory acuity is controlled [42]. Additionally, it is worth pointing out that both the linguistic and musical conditions involved contour pitches, whereas all three control conditions involved pairs of singleton stimulus tokens – as such, individual differences in working memory capacity and sequencing ability may have been differentially implicated in these tasks.

These results contribute to a growing literature on the similarities in processing music and language, especially with respect to pitch perception. These data suggest that individuals exhibit corresponding abilities for pitch perception in both language and music not only because these tasks draw on shared general-purpose attention and working memory processes, and not only because pre-attentive pitch signals are encoded in the same subcortical sensory pathways, but also because there presumably exist higher-level cognitive mechanisms (as yet undetermined) that are shared in the processing of this signal dimension across domains. Through the continued investigation of the relationships among complex and putatively uniquely human cognitive capacities like language and music, we may gain insight into the exaptation processes by which these remarkable faculties evolved [12].

## Supporting Information

Archive S1 **Participant Background Questionnaire and Data.** This archive (.zip) contains a copy of the self-report music and language background instrument (Portable Document Format, .pdf) and participants' summarized responses (OpenDocument Spreadsheet, .ods).
(ZIP)

Archive S2 **Stimulus Generation Scripts.** This archive (.zip) contains the Praat scripts used to generate auditory stimuli and the MATLAB scripts used to generate visual stimuli. All scripts (.praat, .m) are plain text files.
(ZIP)

Archive S3 **Stimulus Files.** This archive (.zip) contains the stimulus files from each condition used in the experiment. Audio files are waveform audio file format (.wav) and image files are bitmap image files (.bmp).
(ZIP)

Archive S4 **Aggregated Participant Behavioral Data.** This archive (.zip) contains two spreadsheets (.ods) summarizing individual participants' performance on the various dependent measures.
(ZIP)
