# Supplementary material for: Evidence for Shared Cognitive Processing of Pitch in Music and Language
Source: PLoS One. 2013 Aug 15;8(8):e73372. doi: 10.1371/journal.pone.0073372 (PMC3744486; doi:10.1371/journal.pone.0073372)
Supplement: Archive S1 — Participant Background Questionnaire and Data. This archive (.zip) contains a copy of the self-report music and language background instrument (Portable Document Format, .pdf) and participants' summarized responses (OpenDocument Spreadsheet, .ods). (ZIP) [file pone.0073372.s001.zip › Participant-Questionairre.pdf]

Participant # ________ Experiment: __________________________

Computer: __________________________ Date: _____________ Time: _____________

Post-Experiment Questionnaire

Date of Birth: ___________________ Sex: _______

1) Are you right-handed ______ or left-handed ______ ?

2) What languages do you speak? Include your native language and any other languages you know or have studied. For each language, indicate your age when you began using this language, your proficiency on a scale from 1 (beginner) to 10 (fluent), and whether you consider it to be your native language.

		Proficiency (scale: 1-10)				
Language	Age	Speaking	Understanding	Reading	Writing	Native

3a) Are there any problems with your hearing that you know of or suspect? Y _____ N _____

3b) If yes, please describe:____

____

4a) Do you have any known or suspected psychological or psychiatric conditions, including any problems with learning, memory, attention, or reading? Y _____ N _____

4b) If yes, please describe: ____

____

5a) Are there any problems with your vision that you know of or suspect? Y _____ N _____

5b) If yes, please describe:____

____

6a) Have you ever played any musical instruments? Y _____ N _____

Participant # ________ Experiment: __________________________

Computer: __________________________ Date: _____________ Time: _____________

6b) If yes, please list the instrument you played, the years you played it, and your proficiency on a scale from 1 (not proficient) to 10 (extremely proficient / professional).

Instrument (e.g., violin)	Dates (e.g., 1996-1997)	Proficiency
__________________________	__________________________	________________
__________________________	__________________________	________________
__________________________	__________________________	________________
__________________________	__________________________	________________

7a) Have you ever sung in a choir? Y _____ N _____

7b) If yes, please indicate the start year and end year (e.g., 1996-1997): __________________________

8a) Have you ever taken music lessons (instrument or voice)? Y _____ N _____

8b) If yes, please list the instrument on which you took lessons, the style of music (e.g., jazz or classical) and the dates you took lessons:

Instrument (e.g., violin/voice)	Style (e.g., jazz)	Dates (e.g., 1996-1997)
__________________________	__________________________	__________________________
__________________________	__________________________	__________________________
__________________________	__________________________	__________________________

9a) Have you ever had any formal training in music theory? Y _____ N _____

9b) If yes, please indicate the start year and end year (e.g., 1996-1997): __________________________

10) Do you have / are you working on a degree in music? Y _____ N _____

11) How much music do you listen to currently (hours per day)? _____________

12) What style(s) of music do you listen to (e.g., classical, rock, jazz)? ______________________________

____

Participant # ________ Experiment: __________________________

Computer: __________________________ Date: _____________ Time: _____________

---

13) Is there anything else you would like to tell us about your musical background?

____

____

____

14) ***Perceived Difficulty***

Instructions: Please indicate the extent to which you agree or disagree with each of the statements below using the following scale:

1 = strongly agree / **2** = agree / **3** = neither agree nor disagree / **4** = disagree / **5** = strongly disagree

Task #:	1	2	3	4	5	6	7
I think that I did well on this task.							
The task was easy.							
I was not motivated to try my best on this task.*							
Small differences were hard for me to notice.							
This experiment was very difficult.							
I was trying my hardest to give the correct answers.							

* We know that these tasks can be boring or hard and sometimes you're not motivated to try your "very best." That's okay. We're working to revise our tasks to make them more interesting, so please just be honest here; it will help us refine our experiments in the future.

15) Do you have any other impressions about the experiment, including strategies you might have used or problems you noticed with the stimuli? What do you think the point of the experiment was? Please tell us any other thoughts or feelings you have about the experiment in general, or any of the tasks in particular.

____

____

____

____

____

____

Thank you very much for helping with our research!
