# Supplementary figures and images for: Evidence for Shared Cognitive Processing of Pitch in Music and Language
Source: PLoS One. 2013 Aug 15;8(8):e73372. doi: 10.1371/journal.pone.0073372 (PMC3744486; doi:10.1371/journal.pone.0073372)

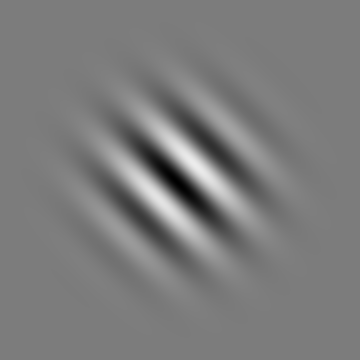

Supplement: Archive S3 — Stimulus Files. This archive (.zip) contains the stimulus files from each condition used in the experiment. Audio files are waveform audio file format (.wav) and image files are bitmap image files (.bmp). (ZIP) [file pone.0073372.s003.zip › Gabors/Gabor_440.bmp]

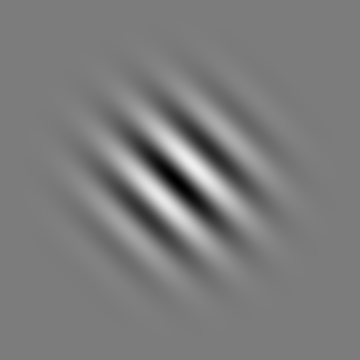

Supplement: Archive S3 — Stimulus Files. This archive (.zip) contains the stimulus files from each condition used in the experiment. Audio files are waveform audio file format (.wav) and image files are bitmap image files (.bmp). (ZIP) [file pone.0073372.s003.zip › Gabors/Gabor_280.bmp]

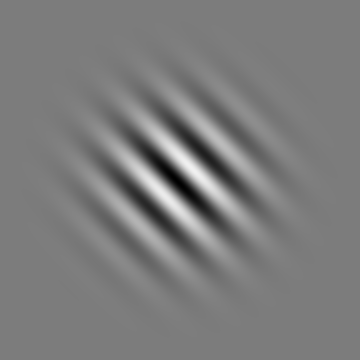

Supplement: Archive S3 — Stimulus Files. This archive (.zip) contains the stimulus files from each condition used in the experiment. Audio files are waveform audio file format (.wav) and image files are bitmap image files (.bmp). (ZIP) [file pone.0073372.s003.zip › Gabors/Gabor_-40.bmp]

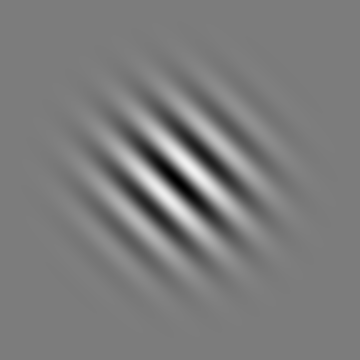

Supplement: Archive S3 — Stimulus Files. This archive (.zip) contains the stimulus files from each condition used in the experiment. Audio files are waveform audio file format (.wav) and image files are bitmap image files (.bmp). (ZIP) [file pone.0073372.s003.zip › Gabors/Gabor_-80.bmp]

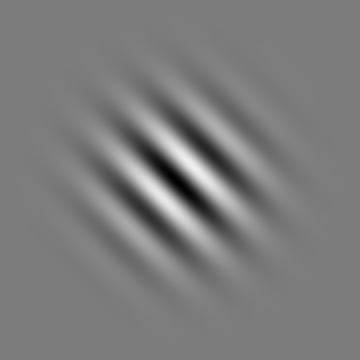

Supplement: Archive S3 — Stimulus Files. This archive (.zip) contains the stimulus files from each condition used in the experiment. Audio files are waveform audio file format (.wav) and image files are bitmap image files (.bmp). (ZIP) [file pone.0073372.s003.zip › Gabors/Gabor_200.bmp]

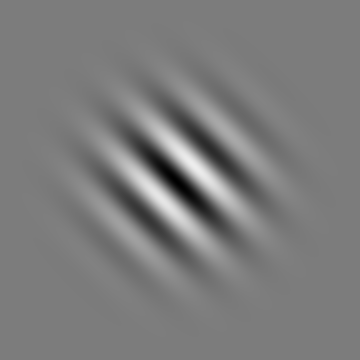

Supplement: Archive S3 — Stimulus Files. This archive (.zip) contains the stimulus files from each condition used in the experiment. Audio files are waveform audio file format (.wav) and image files are bitmap image files (.bmp). (ZIP) [file pone.0073372.s003.zip › Gabors/Gabor_320.bmp]

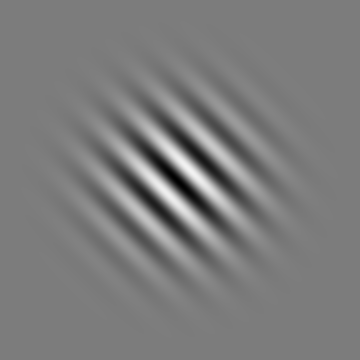

Supplement: Archive S3 — Stimulus Files. This archive (.zip) contains the stimulus files from each condition used in the experiment. Audio files are waveform audio file format (.wav) and image files are bitmap image files (.bmp). (ZIP) [file pone.0073372.s003.zip › Gabors/Gabor_-320.bmp]

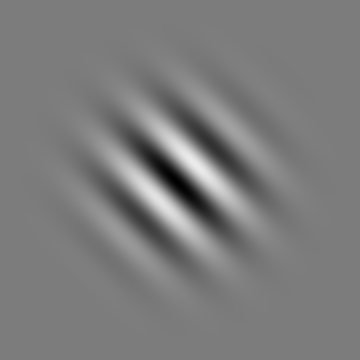

Supplement: Archive S3 — Stimulus Files. This archive (.zip) contains the stimulus files from each condition used in the experiment. Audio files are waveform audio file format (.wav) and image files are bitmap image files (.bmp). (ZIP) [file pone.0073372.s003.zip › Gabors/Gabor_480.bmp]

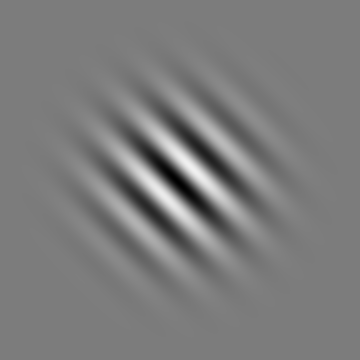

Supplement: Archive S3 — Stimulus Files. This archive (.zip) contains the stimulus files from each condition used in the experiment. Audio files are waveform audio file format (.wav) and image files are bitmap image files (.bmp). (ZIP) [file pone.0073372.s003.zip › Gabors/Gabor_0.bmp]

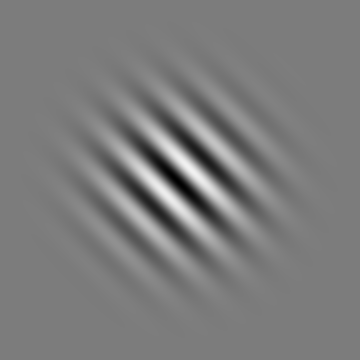

Supplement: Archive S3 — Stimulus Files. This archive (.zip) contains the stimulus files from each condition used in the experiment. Audio files are waveform audio file format (.wav) and image files are bitmap image files (.bmp). (ZIP) [file pone.0073372.s003.zip › Gabors/Gabor_-200.bmp]

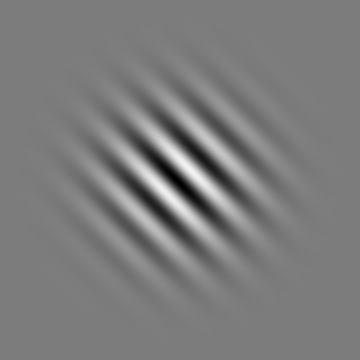

Supplement: Archive S3 — Stimulus Files. This archive (.zip) contains the stimulus files from each condition used in the experiment. Audio files are waveform audio file format (.wav) and image files are bitmap image files (.bmp). (ZIP) [file pone.0073372.s003.zip › Gabors/Gabor_-280.bmp]

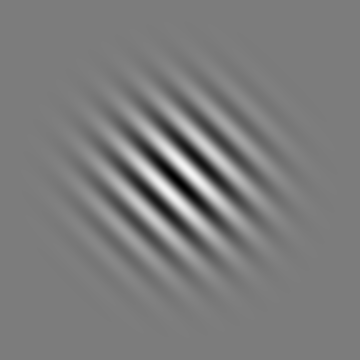

Supplement: Archive S3 — Stimulus Files. This archive (.zip) contains the stimulus files from each condition used in the experiment. Audio files are waveform audio file format (.wav) and image files are bitmap image files (.bmp). (ZIP) [file pone.0073372.s003.zip › Gabors/Gabor_-520.bmp]

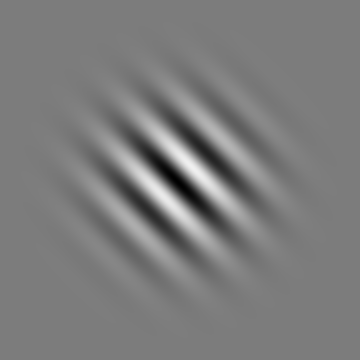

Supplement: Archive S3 — Stimulus Files. This archive (.zip) contains the stimulus files from each condition used in the experiment. Audio files are waveform audio file format (.wav) and image files are bitmap image files (.bmp). (ZIP) [file pone.0073372.s003.zip › Gabors/Gabor_80.bmp]

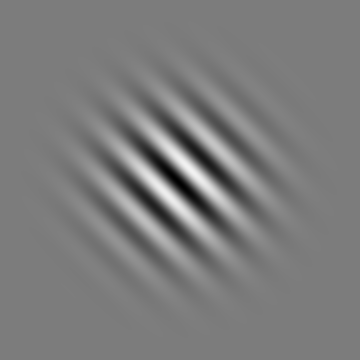

Supplement: Archive S3 — Stimulus Files. This archive (.zip) contains the stimulus files from each condition used in the experiment. Audio files are waveform audio file format (.wav) and image files are bitmap image files (.bmp). (ZIP) [file pone.0073372.s003.zip › Gabors/Gabor_-240.bmp]

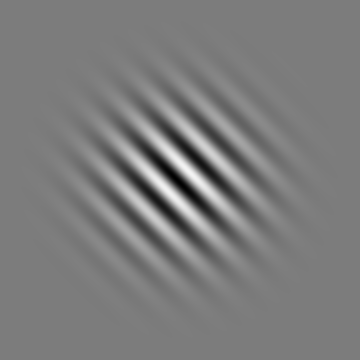

Supplement: Archive S3 — Stimulus Files. This archive (.zip) contains the stimulus files from each condition used in the experiment. Audio files are waveform audio file format (.wav) and image files are bitmap image files (.bmp). (ZIP) [file pone.0073372.s003.zip › Gabors/Gabor_-560.bmp]

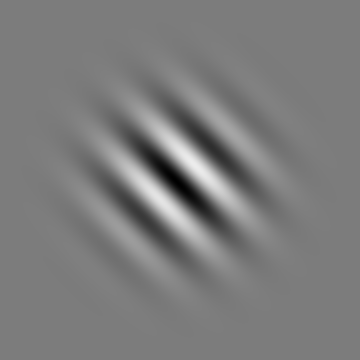

Supplement: Archive S3 — Stimulus Files. This archive (.zip) contains the stimulus files from each condition used in the experiment. Audio files are waveform audio file format (.wav) and image files are bitmap image files (.bmp). (ZIP) [file pone.0073372.s003.zip › Gabors/Gabor_400.bmp]

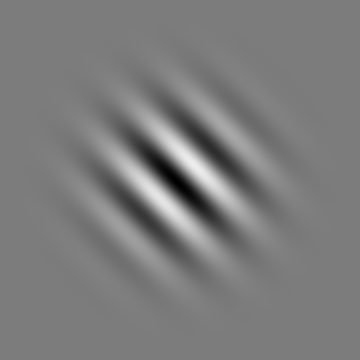

Supplement: Archive S3 — Stimulus Files. This archive (.zip) contains the stimulus files from each condition used in the experiment. Audio files are waveform audio file format (.wav) and image files are bitmap image files (.bmp). (ZIP) [file pone.0073372.s003.zip › Gabors/Gabor_360.bmp]

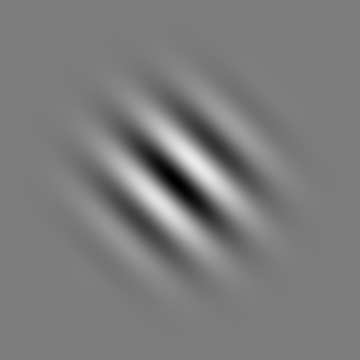

Supplement: Archive S3 — Stimulus Files. This archive (.zip) contains the stimulus files from each condition used in the experiment. Audio files are waveform audio file format (.wav) and image files are bitmap image files (.bmp). (ZIP) [file pone.0073372.s003.zip › Gabors/Gabor_560.bmp]

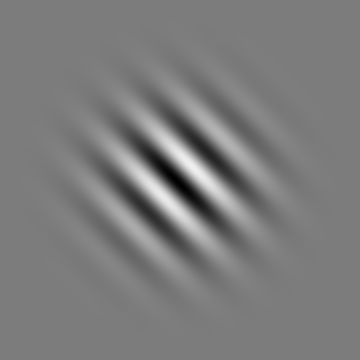

Supplement: Archive S3 — Stimulus Files. This archive (.zip) contains the stimulus files from each condition used in the experiment. Audio files are waveform audio file format (.wav) and image files are bitmap image files (.bmp). (ZIP) [file pone.0073372.s003.zip › Gabors/Gabor_120.bmp]

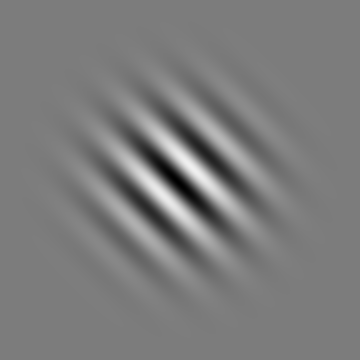

Supplement: Archive S3 — Stimulus Files. This archive (.zip) contains the stimulus files from each condition used in the experiment. Audio files are waveform audio file format (.wav) and image files are bitmap image files (.bmp). (ZIP) [file pone.0073372.s003.zip › Gabors/Gabor_40.bmp]

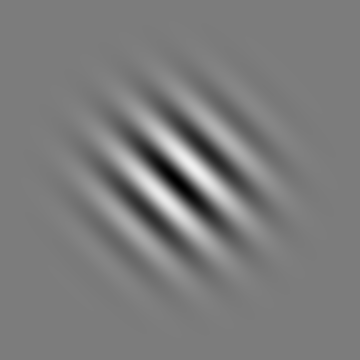

Supplement: Archive S3 — Stimulus Files. This archive (.zip) contains the stimulus files from each condition used in the experiment. Audio files are waveform audio file format (.wav) and image files are bitmap image files (.bmp). (ZIP) [file pone.0073372.s003.zip › Gabors/Gabor_160.bmp]

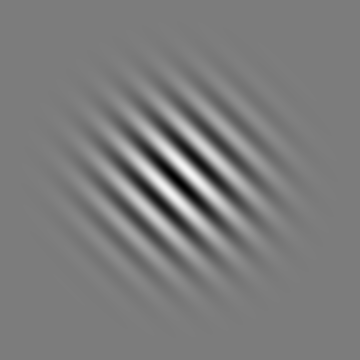

Supplement: Archive S3 — Stimulus Files. This archive (.zip) contains the stimulus files from each condition used in the experiment. Audio files are waveform audio file format (.wav) and image files are bitmap image files (.bmp). (ZIP) [file pone.0073372.s003.zip › Gabors/Gabor_-600.bmp]

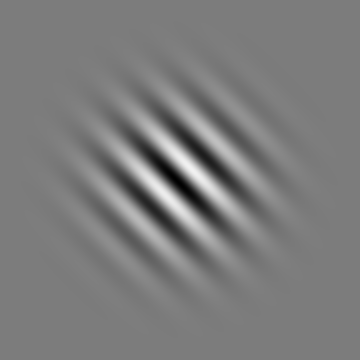

Supplement: Archive S3 — Stimulus Files. This archive (.zip) contains the stimulus files from each condition used in the experiment. Audio files are waveform audio file format (.wav) and image files are bitmap image files (.bmp). (ZIP) [file pone.0073372.s003.zip › Gabors/Gabor_-120.bmp]

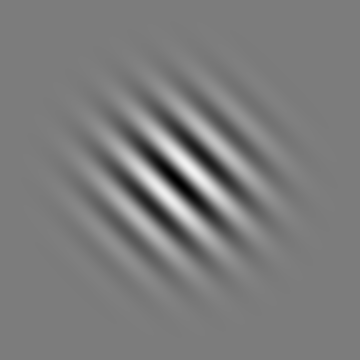

Supplement: Archive S3 — Stimulus Files. This archive (.zip) contains the stimulus files from each condition used in the experiment. Audio files are waveform audio file format (.wav) and image files are bitmap image files (.bmp). (ZIP) [file pone.0073372.s003.zip › Gabors/Gabor_-160.bmp]

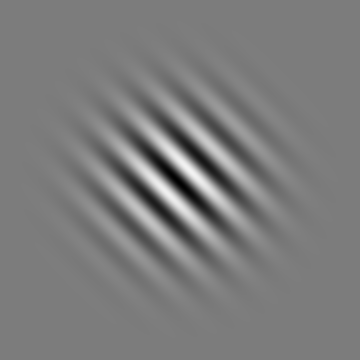

Supplement: Archive S3 — Stimulus Files. This archive (.zip) contains the stimulus files from each condition used in the experiment. Audio files are waveform audio file format (.wav) and image files are bitmap image files (.bmp). (ZIP) [file pone.0073372.s003.zip › Gabors/Gabor_-360.bmp]

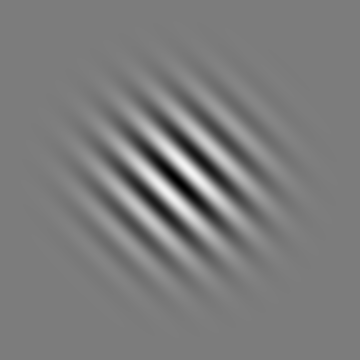

Supplement: Archive S3 — Stimulus Files. This archive (.zip) contains the stimulus files from each condition used in the experiment. Audio files are waveform audio file format (.wav) and image files are bitmap image files (.bmp). (ZIP) [file pone.0073372.s003.zip › Gabors/Gabor_-400.bmp]

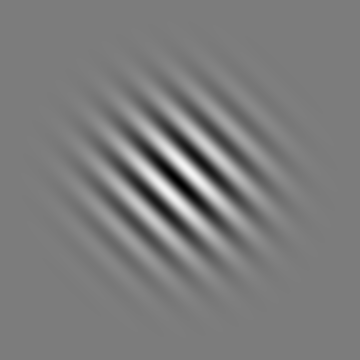

Supplement: Archive S3 — Stimulus Files. This archive (.zip) contains the stimulus files from each condition used in the experiment. Audio files are waveform audio file format (.wav) and image files are bitmap image files (.bmp). (ZIP) [file pone.0073372.s003.zip › Gabors/Gabor_-480.bmp]

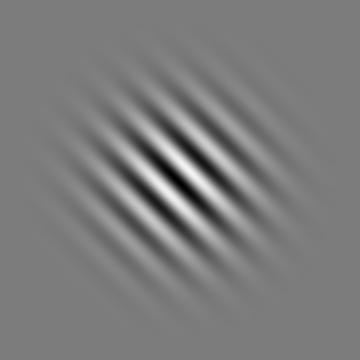

Supplement: Archive S3 — Stimulus Files. This archive (.zip) contains the stimulus files from each condition used in the experiment. Audio files are waveform audio file format (.wav) and image files are bitmap image files (.bmp). (ZIP) [file pone.0073372.s003.zip › Gabors/Gabor_-440.bmp]

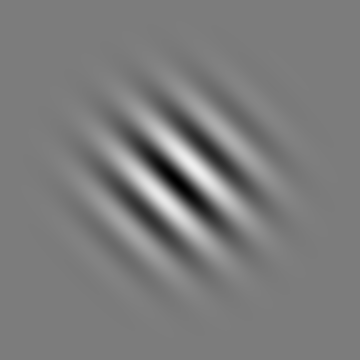

Supplement: Archive S3 — Stimulus Files. This archive (.zip) contains the stimulus files from each condition used in the experiment. Audio files are waveform audio file format (.wav) and image files are bitmap image files (.bmp). (ZIP) [file pone.0073372.s003.zip › Gabors/Gabor_240.bmp]

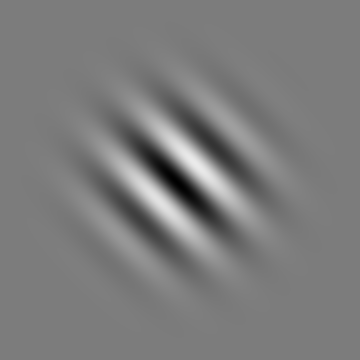

Supplement: Archive S3 — Stimulus Files. This archive (.zip) contains the stimulus files from each condition used in the experiment. Audio files are waveform audio file format (.wav) and image files are bitmap image files (.bmp). (ZIP) [file pone.0073372.s003.zip › Gabors/Gabor_520.bmp]

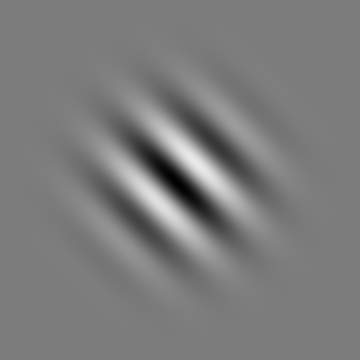

Supplement: Archive S3 — Stimulus Files. This archive (.zip) contains the stimulus files from each condition used in the experiment. Audio files are waveform audio file format (.wav) and image files are bitmap image files (.bmp). (ZIP) [file pone.0073372.s003.zip › Gabors/Gabor_600.bmp]
